# Association of *Helicobacter pylori* and iNOS Production by Macrophages and Lymphocytes in the Gastric Mucosa in Chronic Gastritis

**DOI:** 10.1155/2014/762514

**Published:** 2014-09-18

**Authors:** Lilia A. Cherdantseva, Oksana V. Potapova, Tatyana V. Sharkova, Yana Yu. Belyaeva, Vyacheslav A. Shkurupiy

**Affiliations:** FSBI Research Center of Clinical and Experimental Medicine, SB RAMS, Timakova Street 2, Novosibirsk 630117, Russia

## Abstract

*Helicobacter pylori* is one of the most common causes of chronic gastritis. With the development of the disease cellular inflammatory infiltrates composed of lymphocytes, plasma cells, and macrophages are formed in epithelium and lamina propria of the stomach. These cells are capable of secreting a number of active substances, including inducible nitric oxide synthase (iNOS). We examined the relationship between *H. pylori* and secretion of iNOS by cells of inflammatory infiltrates in chronic gastritis by light microscopy and immunohistochemistry. The data obtained indicate that stimulation of *H. pylori* immune system cells of the host organism during development of chronic gastritis causes increase in number of macrophages and lymphocytes in the inflammatory infiltrate of the gastric mucosa. This is accompanied with increased expression of inducible NO-synthase with excess free radicals in the tissues, which leads to secondary alterations and exacerbates the inflammation with impaired regeneration processes.

## 1. Introduction

*Helicobacter pylori* is one of the most common causes of chronic gastritis. The global human population gets infected by* H. pylori* as early as in childhood and adolescence. Chronic* H. pylori*-associated gastritis develops in more than 50% of infected people [1].* H. pylori* has been proved to be the etiological factor of type B chronic gastritis, gastric and duodenal ulcer, and other gastrointestinal diseases associated with the morphological changes of gastric mucosa and such dysregenerative manifestations as atrophy, metaplasia, and dysplasia underlying neoplastic processes [2].

It is known that inflammatory cellular infiltrate, containing mainly lymphocytes, plasmocytes, and macrophages, is generated in epithelium and lamina propria of the stomach during the development of chronic gastritis, including chronic* H. pylori*-associated gastritis [3]. Lymphocytes, plasmocytes, and macrophages cause the cytokine damage of gastric mucosa with the inducible NO-synthase (iNOS) being a mixed factor [4].

The* Н. pylori* antigens can induce iNOS expression by macrophages and lymphocytes of inflammatory cellular infiltrate in chronic gastrointestinal conditions. Urease,* Н. pylori* pathogenicity factor, can directly inhibit the phagocytic activity of macrophages according to the literature data [5]. Urease can influence the level of iNOS expression by inflammatory infiltrate cells and the accumulation of nitrogen oxide and thereby regulate the inflammatory process [6–8]. The iNOS expression in chronic* H. pylori*-associated gastritis is also induced by bacterial outer membrane lipopolysaccharides that possess antigen properties and induce host antibacterial response and destructive changes in gastric mucosa [9].

However, contemporary literature lacks the data on the role of lymphocytes and macrophages in oxygen-dependent mechanisms of protection from* H. pylori* infection at the tissue and cellular levels, obtained by gastric mucosa biopsies study. Aforesaid the purpose of the current study was to investigate the* Н. pylori*-induced iNOS expression by lymphocytes and macrophages of gastric mucosa in chronic gastritis.

## 2. Materials and Methods

For this investigation we used paraffin-embedded antrum biopsies from the archive of the clinic of Research Center of Clinical and Experimental Medicine (Novosibirsk, Russia). Tissue samples were obtained at endoscopy with biopsy gastric antral mucosa from patients with a first diagnosed chronic gastritis in 2009–2013. The urease test (Jatrox-H.p.-Test, Germany) was used to detect* H. pylori* in tissue samples indirectly.

Sections of 3-micron thickness were prepared on a rotary microtome HM355S (“Microm”, Germany) and stained with hematoxylin and eosin by standard procedure to determine the severity and activity of chronic gastritis; light microscopy standard techniques were used. For* H. pylori* visualization Giemsa stain technique was used. Morphological assessment of biopsies was performed by visual analogue scale in accordance with the “Sydney system” and the classification of chronic gastritis described by Dixon et al. [10] and Aruin et al. [9] with a semiquantitative assessment of the degree of contamination of the gastric mucosa* H. pylori*.

After preliminary histological evaluation two study groups were formed. The first group (62 biopsy specimens) were patients with chronic moderate* H. pylori*-associated gastritis with moderate activity and low degree of bacterial contamination (*H. pylori* +). The average age of patients in this group was 56 years. The second group (56 biopsy specimens) consisted of patients with chronic moderate* H. pylori*-negative gastritis with moderate activity and an average age of 58 years.

Immunohistochemical (IHC) analysis was performed by using indirect streptavidin-peroxidase method with specific primary antibodies against inducible nitric oxide synthase (iNOS, “Spring BioScience”) and macrophage marker CD68 (“DBS”). To visualize the antibodies “NovoLink” detection system (“Novocastra”) was used. For IHC studies sections were dewaxed and rehydrated. After antigen unmasking in a microwave oven at 700 W power for 20–25 minutes and washing with distilled water, phosphate buffer, endogenous peroxidase was blocked within 5 minutes. Exposure time to the primary antibodies was 30–45 minutes at 37°C. Sections were incubated with streptavidin-peroxidase complex and DAB-substrate and were further counterstained with Mayer's hematoxylin.

Morphometric study of tissue structural elements was conducted using closed test system consisting of 100 points, square 3.6 × 10^5^ *μ*m^2^. There were registered volume density (Vv) of inflammatory infiltrates in the lamina propria and the numerical density (Nai) of lymphocytes, plasmocytes, and CD68+ macrophages and cells expressing iNOS [11]. Statistical analysis of the results was performed using the statistical analysis package Microsoft Office Excel 2007 and standard software package STATISTICA v.6. The arithmetic mean value (M) and standard error of the mean (m) were determined. To identify the probability of significance of differences of compared average values Student's *t*-test was used. Differences were considered statistically significant at the 5% significance level (*P* < 0.05).

## 3. Results

**Figure 1:**
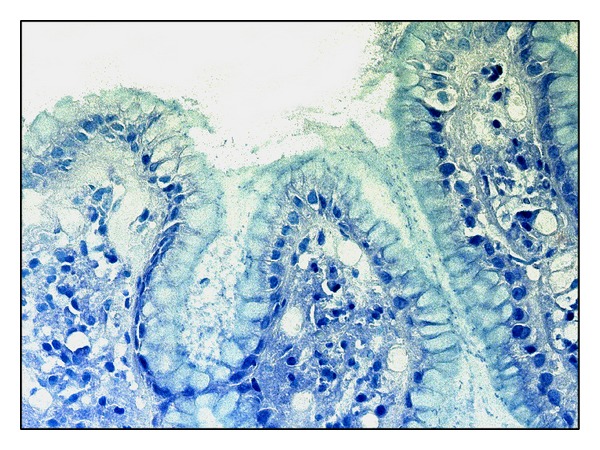
Antrum mucosa in* H. pylori*-associated gastritis: mucus masses with* Н. pylori* agglomerations on mucosa surface, Giemsa staining, magnitude ×200.

**Figure 2:**
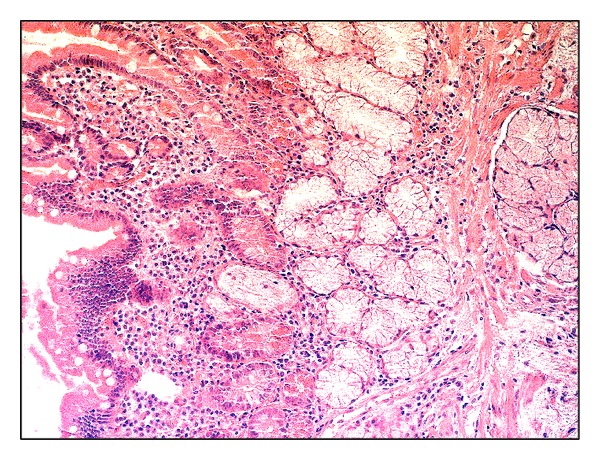
Antrum mucosa in* H. pylori*-associated gastritis: focal enteric metaplasia of epithelium, the lymphocytoplasmocytic infiltration of lamina propria with the admixture of neutrophils, focal fibrosis, hematoxylin and eosin staining, magnitude ×200.

Signs of moderate chronic gastritis with moderate activity and low level of* Н. pylori* contamination (+) were detected in the first study group using the general light microscopy of antrum biopsy material histological sections (Figure 1). Gastric mucosa represented a mature hypersecretory epithelium with erosions, sites of foveolar hyperplasia, and focal enteric metaplasia of foveolar epithelium. There were a mild edema, focal lymphocytoplasmocytic infiltration with more than 50% proportion of plasmocytes, and the admixture of neutrophils and a focal fibrosis in lamina propria (Figure 2).

Signs of moderate chronic gastritis with moderate activity and no signs of* Н. pylori* contamination (−) were detected in the second study group. Gastric mucosa represented a mature epithelium with sites of enteric metaplasia of foveolar epithelium. Moderate lymphocytoplasmocytic infiltration with more than 60% proportion of plasmocytes and the admixture of neutrophils and small fibrosis foci were detected in lamina propria.

**Figure 3:**
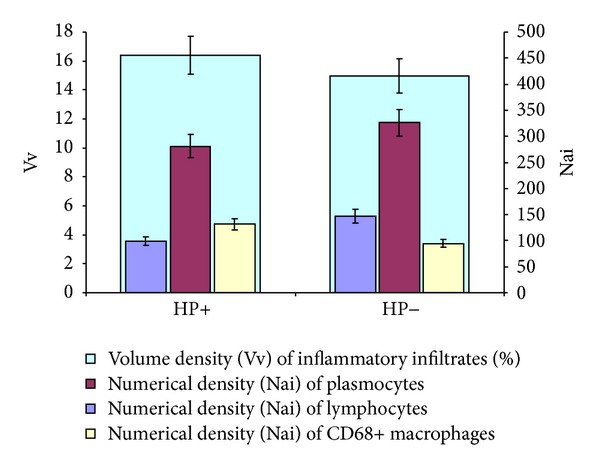
Volume density (Vv) of inflammatory infiltrates and numerical density (Nai) of lymphocytes, plasmocytes, and CD68+ macrophages study results.

**Figure 4:**
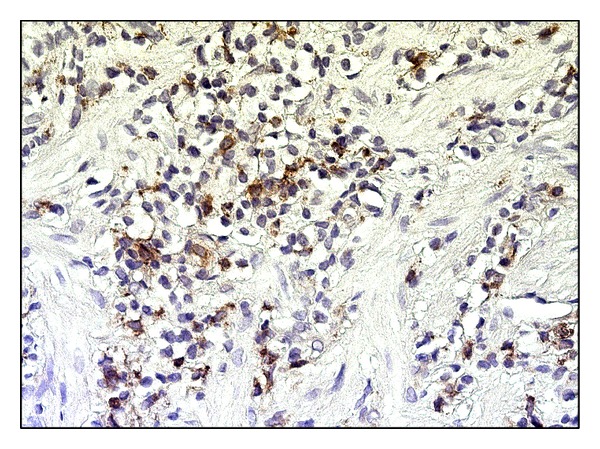
Antrum mucosa in* H. pylori*-associated gastritis: CD68+ macrophages in infiltrate of lamina propria, magnitude ×400.

The morphometric study of histological sections in both groups has not revealed significant differences between the values of volume density of inflammatory infiltrates in lamina propria (Figure 3). The numerical density of lymphocytes in inflammatory infiltrate of gastric lamina propria in the second group was 1.5-fold higher than in the first group (Figure 3). Large number of CD68+ macrophages was detected in gastric mucosal biopsy material in the first study group. The numerical density in the first group was 1.4-fold higher than in the second group (Figures 3 and 4).

**Figure 5:**
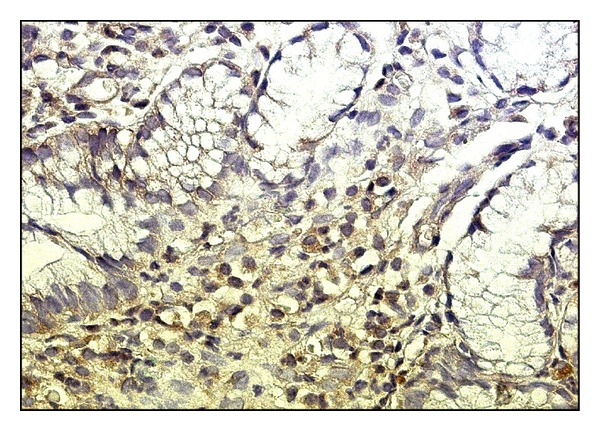
Antrum mucosa in* H. pylori*-associated gastritis: the iNOS expression by macrophages and lymphocytes of inflammatory infiltrate in lamina propria, magnitude ×400.

**Figure 6:**
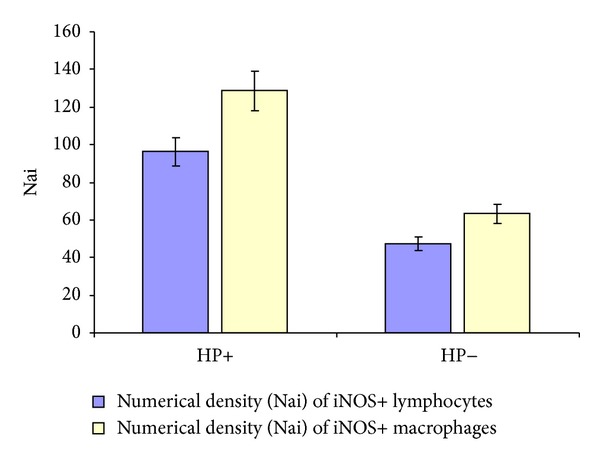
The numerical density (Nai) of lymphocytes and macrophages expressing iNOS.

Numerical densities of iNOS+ lymphocytes and iNOS+ macrophages in the first study group (Figure 5) were 2-fold higher than those in the second study group (Figure 6). A 1.3-fold higher numerical density of iNOS+ macrophages in comparison with iNOS+ lymphocytes was noted in both groups. Thus the number of iNOS+ cells was significantly higher in antrum mucosa in case of chronic* H. pylori*-associated gastritis with low level of bacterial contamination than in case of chronic* H. pylori*-negative gastritis (Figure 6).

## 4. Discussion

Currently cytokines,* Н. pylori* antigens, and its pathogenicity gene cluster are considered among the pathogenicity factors of* Н. pylori*. Their activation launches a number of pathogenic mechanisms of gastric mucosal inflammation associated with destruction on molecular, cellular, and tissue level and with dysregenerative manifestations [12].

The results of this study suggest that the volume density of the inflammatory infiltrates in groups 1 and 2 did not have significant differences. However, the presence of* H. pylori* in the gastric mucosa had a significant effect on the cellular composition of infiltrates that exhibits a decrease in the number of lymphocytes and an increased number of macrophages in group 1 compared to group 2.

An activation of nuclear transcription factor NF-*κ*В in epithelial cells and neutrophils of gastric mucosa during their interaction with CagA protein of* Н. pylori* outer membrane is a key moment of inflammation initiation that results in the release of many proinflammatory cytokines [13]. Literature data suggest that during the chronization of inflammation these cytokines support the chemotaxis and chemokinesis of leucocytes and macrophages with an increase of their numbers in inflammation area [3, 14, 15].

It is known that the migration of leucocytes and macrophages to inflammation area is associated with generation of active oxygen forms and cell destruction with the release of cytotoxic enzymes determining the destructive changes in gastric mucosa [9]. Inflammatory process in gastrointestinal tissues is also associated with an increase of secretory activity of lymphocytes and macrophages. Proinflammatory cytokines production can be accompanied with the iNOS expression by inflammatory infiltrate cells [16–18].

The number of lymphocytes and macrophages expressing iNOS in antrum mucosa was calculated to evaluate the inducible NO-synthase expression at the tissue and cellular levels. The data obtained showed that contamination of the gastric mucosa by* H. pylori* leads to activation of effector cells of the immune system of the host organism manifesting twofold increased number of macrophages and lymphocytes expressing an inducible form of NO-synthase.

The inducible NO-synthase is associated with the production of NO that is the factor of oxygen-dependent system of antiviral and antibacterial protection [19]. However, the overaccumulation of reactive oxygen metabolites in tissues causes the toxic effect on tissue cells, severe destructive changes, and dysregenerative disorders. This is consistent with more significant destructive changes of gastric mucosa, erosions, and such regeneration disorder as a focal enteric metaplasia detected in histological study of* H. pylori*-associated gastritis.

## 5. Conclusion

The data obtained as a result of histological examination of the gastric mucosa at the tissue and cellular levels indicate that* H. pylori* stimulation of immune cells of the host organism during development of chronic gastritis causes increase in number of macrophages and lymphocytes in the inflammatory infiltration of gastric mucosa with the activation of their functional activity, including oxygen-dependent mechanisms of immune response. It is associated with an increased macrophage and lymphocyte expression of inducible NO-synthase and the overaccumulation of free radicals in tissues leading to the secondary alteration, irrespective of etiological factor presence and intensity, and promotes persistence and aggravation of inflammatory process with dysregenerative manifestations.
